# Factors associated with low fitness in adolescents – A mixed methods study

**DOI:** 10.1186/1471-2458-14-764

**Published:** 2014-07-29

**Authors:** Richard Charlton, Michael B Gravenor, Anwen Rees, Gareth Knox, Rebecca Hill, Muhammad A Rahman, Kerina Jones, Danielle Christian, Julien S Baker, Gareth Stratton, Sinead Brophy

**Affiliations:** College of Medicine, Swansea University, Swansea, SA2 8PP UK; School of Sport, University of Wales Institute Cardiff, Cardiff, CF23 6XD UK; Applied Sport Science University of West of England (Hartpury College), Gloucester, England; College of Health and Human Sciences, Swansea University, Swansea, SA2 9PP UK; Institute of Clinical Exercise and Health Science, School of Science, University of the West of Scotland, Hamilton, Lanarkshire, ML3 OJB Scotland; College of Engineering, Swansea University, Swansea, SA2 8PP UK

**Keywords:** Eduation and health, Risk factors, Diabetes, Heart disease, Physical activity

## Abstract

**Background:**

Fitness and physical activity are important for cardiovascular and mental health but activity and fitness levels are declining especially in adolescents and among girls. This study examines clustering of factors associated with low fitness in adolescents in order to best target public health interventions for young people.

**Methods:**

1147 children were assessed for fitness, had blood samples, anthropometric measures and all data were linked with routine electronic data to examine educational achievement, deprivation and health service usage. Factors associated with fitness were examined using logistic regression, conditional trees and data mining cluster analysis. Focus groups were conducted with children in a deprived school to examine barriers and facilitators to activity for children in a deprived community.

**Results:**

Unfit adolescents are more likely to be deprived, female, have obesity in the family and not achieve in education. There were 3 main clusters for risk of future heart disease/diabetes (high cholesterol/insulin); children at low risk (not obese, fit, achieving in education), children ‘visibly at risk’ (overweight, unfit, many hospital/GP visits) and ‘invisibly at risk’ (unfit but not overweight, failing in academic achievement). Qualitative findings show barriers to physical activity include cost, poor access to activity, lack of core physical literacy skills and limited family support.

**Conclusions:**

Low fitness in the non-obese child can reveal a hidden group who have high risk factors for heart disease and diabetes but may not be identified as they are normal weight. In deprived communities low fitness is associated with non-achievement in education but in non-deprived communities low fitness is associated with female gender. Interventions need to target deprived families and schools in deprived areas with community wide campaigns.

## Background

Fitness and physical activity are important for health, growth and development during childhood and adolescence [1, 2]. Adolescence is a critical period for the acquisition of health-related behaviours and behaviours learned in childhood are known to track into adulthood [3, 4]. However, physical inactivity is now a major public health problem reportedly responsible for 9% of premature mortality worldwide in 2008 [2]. Despite the health benefits of physical activity [5] there has been evidence of decreasing levels in recent decades [6, 7]. Likewise, evidence shows that fitness has been declining over recent decades [8]. Poor fitness is associated with increased risk of cardiovascular disease in children [9, 10]. Despite a growing emphasis on the importance of physical activity and physical fitness in childhood and adolescence, factors promoting fitness remain unclear. Physical activity is a multi-factorial behaviour influenced by psychological, social, environmental and demographic variables. The prevalence of adolescents not meeting the current physical activity guidelines has been estimated at 80.3% [11]. Research to date has shown that socio-economically deprived, ethnic minority children and girls have lower physical activity rates [7, 12] and that activity declines with age [13, 14]. Active travel to school and opportunities for physically active play are declining [13, 15] and sedentary activities are increasing [16].

Given strong evidence of fitness as a critical marker of adolescent and adult health [17], it is desirable to formulate effective and timely preventive strategies starting early in life. To achieve this, we need first to identify those factors associated with fitness during adolescence. The aim of this paper is to examine clustering of risk factors in order to highlight where public health interventions might be most effective and to explore the barriers to activity among teenagers from a deprived background so that public health intervention can be targeted to address these barriers.

## Methods

The detailed methods of this cross sectional survey have previously been described in a protocol paper and prevalence paper [7, 18].

### Recruitment and data collection

The study population was recruited from ten schools in south Wales (UK). Schools were selected according to deprivation of the catchment area in that half were selected as being in deprived areas (in order of most deprived selected first) and half as having a non deprived catchment (largest schools selected first). All children in years 7 and 8 (i.e. aged 11 to 13 years) were eligible. Data collection occurred during the school year 2009/10. All testing procedures took place on school premises, and during allocated physical education lessons. The data collected included; demographic data, anthropometric and physiological data such as blood samples for fasting lipids and glucose, physical activity and dietary intake. In addition, children and parents completed a questionnaire detailing family history of diseases, parental BMI, birth weight and general health.

#### Fitness

Aerobic fitness was measured using the 20 metre multi stage fitness test (20MSFT) [19], an incremental running test that has proven to be a valid and reliable method of assessing aerobic fitness in young people. Children were classed as unfit if they performed less than level 6.8 (49 shuttles) for boys, and less than level 4.5 (27 shuttles) for girls [20]. The 20MSFT is used routinely in physical education lessions in schools so all the children (participants and non-participants) had fitness assessment as part of their routine school day.

#### Anthropometric and physiological data

Anthropometric data collected included stature, body mass (BMI – Body mass index), skinfold thickness, neck, waist and hip circumference, and blood pressure. Body weight was recorded to the nearest 0.1 kg using calibrated electronic weighing scales (Seca 770, Digital Scales, Seca Ltd, Birmingham, UK). Stature was measured using a portable stadiometer (Seca Stadiometer, Seca Ltd, Birmingham, UK). Body mass index (BMI) was calculated using the BMI formula of dividing the participants’ weight by their height squaredand participants were categorised as overweight or obese as defined by the International Obesity Task Force (IOTF) age and sex specific cut-offs for BMI [21]. Systolic and diastolic blood pressure (BP) were measured using an Omron M6 automatic BP monitor (Omron Healthcare UK Ltd, Milton Keynes, UK). Blood-pressure was recorded three times, with the average of the second and third reading recorded for data analysis. Children were classified as having high blood pressure if the Systolic was >130 mm Hg or the diastolic was >85 mm Hg [22, 23].

#### Measures of deprivation and ethnicity

The school was asked to complete a form providing information on the number of children receiving free school meals, thus identifying the deprivation level of the school [24]. Free school meals are allocated to pupils based on family income levels and are a marker of the individual child’s deprivation level. Schools were classified as being situated in a deprived catchment area if more than 21% of the school children were eligible to receive free school meals. This is an arbitrary cut off based on being above the Welsh average of 15% (95% CI: 14.8%- 15.2%) of secondary school children being eligible for free school meals [25]. The deprived schools also scored highly on the Multiple Index of Deprivation [26], a measure based on levels of child poverty, unemployment, health deprivation and disability. The school records were used to identify the free school meal eligibility for each child and this was used as a marker for individual child deprivation level. Therefore, we had an assessment of the deprivation level of the school based on the proportion of children in the school recieving free school meals, and we have an assessment of the individual childs deprivation based on their person eligablity for free school meals. Each child was also asked to self report their ethnicity.

#### Lipids and lipoproteins

Venous blood samples were taken from each consenting participant. These were taken first thing in the morning whilst the children were in a fasted state. Blood samples were taken by qualified phlebotomists, with a nurse or doctor present at all times. Blood samples were analysed for fasting levels of glucose, insulin, lipids, high molecular weight adiponectin and high sensitivity C-reactive protein (CRP). Age and gender specific cut off points proposed by the International Diabetes Federation (IDF) [27] were used for the biochemical risk factors of high triglyceride (≥1.7 mmol/L), low levels of high density lipoprotein cholesterol (<1.03 mmol/L), total cholesterol/High density lipoprotein ratio of >4, and elevated blood glucose (≥5.6 mmol/L) [27, 28].

#### Questionnaire on family health

Parents and children were asked to complete a questionnaire regarding family history of conditions, parents’ height and weight, and ethnic background. However, this questionnaire was only introduced after the first 4 schools had been recruited and participated. Therefore, not all children and parents were given this background questionnaire.

#### Ethical approval

Ethics approval for this study was granted by the Local NHS Research Ethics Committee - Dyfed Powys REC. Written parent and child consent was gained for each participant.

### Routine data

The results of the health survey were linked with electronic records of the National Child Health Database, educational records, GP visits and vaccinations, and attendance at the emergency department. This was done through the a Secure Anoynimised linkage system [29, 30]. The National Child Health Database provides information from pregnancy and birth such as birth weight, gestational age, mothers smoking status (in pregnancy), and vaccinations. The educational records provides information about formally assessed tests such as the Key stage achievement tests. These assess the level of achievment in English, Maths, and other core subjects and assesses if the child has reached the recommended level for their age. Key stage 1 is assessed at age 7–8 years, key stage 2 is at 10–11 years and Key stage 3 is at age 14–15. The GP and attendance at emergency departments are recorded as date of attendence and code associated with that attendence (e.g. diagnosis, medication, symptom, procedure etc.).

#### Focus groups

Qualitative analysis using four focus groups were held between January and June 2012 with 20 children (aged 13–14, 2 groups of girls (n = 10) and 2 groups of boys (n = 10)) who were classified as deprived and who attended a deprived school Sampling was purposeful to ensure equal numbers of boys and girls were included. Of groups eligible, the children were chosen at random and parental consent was obtained. Groups were run within school during school time and lasted 1 hour. Children were asked about their physical activity and perceived barriers to and motivators for engagement in activity. All focus groups were tape recorded with permission and transcribed verbatim. Two researchers were present at each group (DC & SB or DC & RH). In addition, one researcher (DC) ran face-to-face interviews with two teachers from one school, namely the Physical Education teacher and the Head of Year for Year 9 (age 13–14). Interviews were conducted on the school premises during the lunch time break. The focus groups and interviews followed a semi-structured format with a grounded theory approach. Each transcript was coded independently by two members of the research team to develop an agreed coding frame which was refined through discussion.

### Statistical analysis

#### Predicting Fitness

Two different methods were used to examine factors associated with fitness; 1. Logistic regression - Each variable was analysed individually using univariate analysis, then all significant variables were combined in a mutually adjusted model (i.e. each variable adjusted for the others). R was used for all analysis and school level variation was adjusted for as a random effect. Therefore, confidence intervals presented represent adjustment for school level variation. 2. Conditional Trees - An automated approach was used to produce a conditional tree to predict fitness. At each node, the data was split according to the most significant variable, and each node was conditional on the node before.

#### Sensitivity analysis

To examine the effect of missing data, multiple imputation was performed in R using the package mice (Multiple Imputations by Chained Equations), which generates multiple imputations by Gibbs sampling.

#### Fitness/Weight

Data was stratified into four categories of 1. ‘Normal fitness, Normal weight’, 2. ‘Normal fitness/Overweight’, 3. ‘Unfit/Normal weight’, 4. ‘Unfit/Overweight’, and descriptive statistics were presented for all explanatory variables.

#### Cluster Analysis to examine clustering of fitness and weight

Intelligent Miner (IM) was used to undertake the cluster analysis as this tool provides fast and natural clustering of very large databases.

## Results

The detailed demographic details of the children participating in the health survey have previously been published [7]. However, of 3029 children invited, 1147 (38%) participated in the study. Of these (490 male; 657 female) a third were overweight, 1 in 6 had elevated blood pressure, more than 1 in 10 had high cholesterol, 58% consumed more fat than recommended. Of the 881 children tested for fitness, 328 were unfit, giving a prevalence of being unfit of 37.2% (95% CI: 34% to 40%, n = 328/881).

Those who participated were comparable to non-participants in terms of cardiovascular fitness as measured by the 20 MSFT (class average level (e.g. average of all children) 5.8 compared to sample average level 6.1). Eleven schools were approached to participate in the study, with one school in a deprived area declining as they did not want to be involved in research. Therefore, 90% of schools approached participated in the research (5 from affluent and 5 from deprived catchement areas). Of the 1147 participating children, 918 (81%) undertook blood sampling; in terms of BMI, there was no difference between those giving blood samples (20.58 kg/m^2^ (stdev: 3.81)) and those who did not (20.38 kg/m^2^ (stdev: 4.24)). There were 84 (7%) children who were of ethnic minority background and 352 (31%) who were overweight or obese. A higher proportion of those in deprived catchment areas did not return a parental assent form and therefore could not participate in the study.

### Predicting fitness

**Figure 1:**
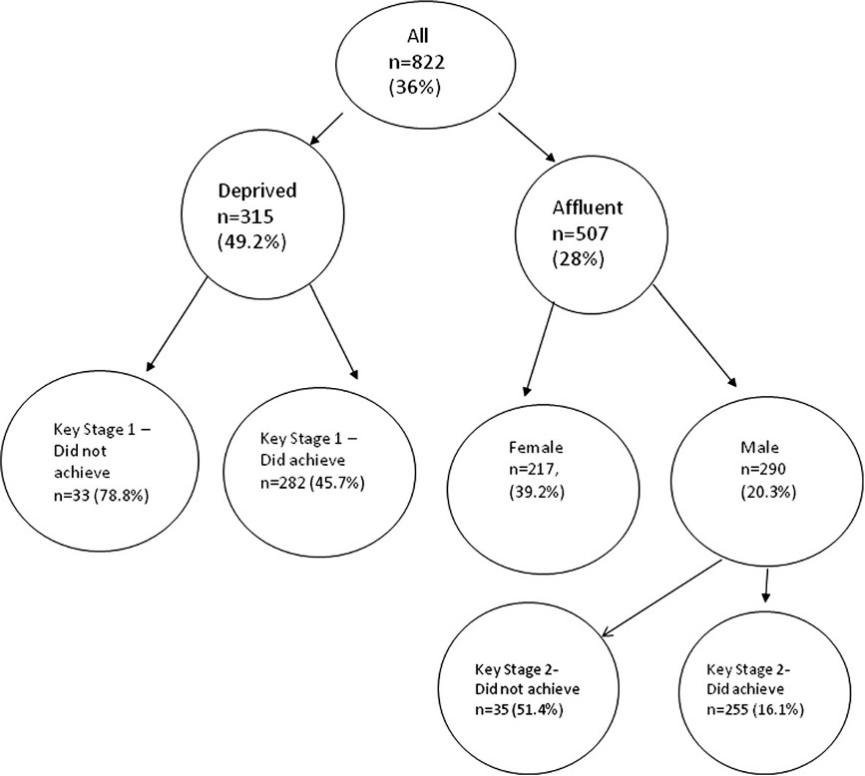
**Conditional Tree.** Percentages are proportion of unfit children in any given node and n is number of children in the node.

Factors associated with unfit/fit are presented in Table 1. Those factors associated with being unfit were entered into a logistic regression model. Deprived catchment area for school (Odds ratio: 2.4 (95% CI: 1.82, 3.28)), female gender (OR: 1.98 (95% CI: 1.48, 2.65)), mother obese (OR: 1.81(95% CI: 1.13,2.92)), not achieving Key Stage 1 (KS1, age 7–8) (OR: 4.00 (95% CI: 2.08, 8.33)), not achieving Key Stage 2 (KS2, age 10–11) (OR 2.44 (95% CI: 1.67, 3.70)) and large in weight in the first 5 years of life (OR: 3.23 (95% CI: 1.74, 6.22)) were all independently associated with being unfit (adjusted for school level variation, n = 822). Poor fitness was associated with metabolic related risk factors including higher cholesterol (16.5% compared to 10.1%, unfit vs fit respectively), triglyceride (0.87 compared to 0.6, unfit vs fit respectively), cholesterol ratio (9.2% compared to 2.1%, unfit vs fit respectively) and higher fasting insulin (11.52 compared to 8.32, unfit vs fit respectively).The conditional tree approach (Figure 1) highlighted Deprivation, Gender, and Education as predictors of fitness. Children in deprived areas who do not achieve their Key stage 1 (age 7–8) were very likely to be unfit at age 11. In non-deprived areas, girls were more likely than boys to be unfit. In non-deprived areas, boys who do not achieve Key Stage 2 (age 10–11) were less likelty to be fit. However, non-deprived girls were more likely to be fit than the children (boys and girls) in a deprived area.

**Table 1:** **Factors associated with being unfit – all variables examined (univariable analysis)**

Variables	Unfit (n = 328)	Fit (n = 553)	Odds (95% CI)
Fitness Predictors			
Deprived/Affluent*	**51.8%**	**30.6%**	**2.44 (1.82, 3.28)**
Female Gender*	**52.8%**	**36.1%**	**1.98 (1.48, 2.65)**
Ethnic Group	14.4%	10.8%	1.39 (0.80, 2.40)
Birth Weight	3.47	3.44	1.11 (0.87, 1.41)
Illness Affecting Exercise	12.6%	8.2%	1.62 (0.95, 2.81)
Receiving Medical Treatment	13.8%	11.9%	1.19 (0.73, 1.94)
Heart Disease In Family	40.7%	38.7%	1.08 (0.74, 1.59)
Diabetes In Family	46.2%	40.7%	1.25 (0.85, 1.83)
Stroke In Family	25.3%	19.4%	1.40 (0.88, 2.24)
High Bp In Family*	**59.7%**	**49.1%**	**1.54 (1.07, 2.22)**
High Chol In Family	42.3%	36.5%	1.27 (0.87, 1.86)
Smoker In Family	47.4%	45.6%	1.08 (0.75, 1.54)
Mother Obese*	**20.3%**	**12.3%**	**1.81 (1.13, 2.92)**
Father Obese	26.7%	22.3%	1.27 (0.83, 1.93)
**Child obese**	30.3%	8.07%	**4.96 (3.36, 7.32)**
Vaccination	48.8%	55.4%	0.77 (0.58, 1.02)
FSM Eligible*	**28.0%**	**18.8%**	**1.67 (1.01, 2.78)**
KS1 Test*	**78.3%**	**93.5%**	**0.25 (0.12, 0.48)**
KS2 Test*	**75.5%**	**88.4%**	**0.41 (0.27, 0.60)**
KS3 Test	75.0%	80.1%	0.75 (0.33, 1.79)
Large for Gest Age	24.2%	21.0%	1.20 (0.85, 1.69)
Large 6 m – 12 m*	**22.5%**	**14.0%**	**1.78 (1.01, 3.15)**
Large 12 m – 5 y*	**21.2%**	**7.7%**	**3.23 (1.74, 6.22)**
**Health Outcomes**			
High BP	12.2%	9.1%	1.37 (0.86, 2.18)
High chol*	**16.5%**	**10.1%**	**1.76 (1.11, 2.76)**
Triglyceride*	**0.87**	**0.69**	**3.72 (2.34, 6.03)**
High Chol:HDLChol Ratio*	**9.2%**	**2.1%**	**4.67 (2.11, 11.09)**
High glucose	3.6%	3.2%	1.11 (0.43, 2.67)
Fasting insulin*	**11.52**	**8.32**	**1.09 (1.05, 1.13)**
CRP	1.24	1.07	1.08 (0.97, 1.20)
Adiponectin	3,878	3,768	1.00 (1.00, 1.00)
Asthma	12.7%	9.2%	1.44 (0.91, 2.26)
Infection	24.7%	23.7%	1.06 (0.76, 1.47)
Tonsillitis	19.7%	22.4%	0.85 (0.60, 1.21)
Mean Visit gp	53.38	49.05	1.00 (1.00, 1.01)
Mean Visit out	2.9	3.32	0.99 (0.96, 1.01)
Mean Visit in	1.26	1.00	1.05 (0.99, 1.14)
Mean Visit ae major	0.37	0.38	0.99 (0.83, 1.17)
Mean Visit ae minor	0.04	0.07	0.74 (0.42, 1.16)

(*denotes difference using 95% CI).

### Sensitivity

Multiple imputations showed no change in findings. Checking for the effect of the missing data, we found that overall the p-values remained almost the same as the p-values of the models before multiple imputations.

### Fitness and weight

Descriptive statistics (Table 2) showed that children who were overweight, even if they were fit, were more likely to have high blood pressure, high HDL/LDL cholesterol ratio, and an obese mother, and to be large in weight at 6 months to 12 months, and at 12 months to 5 years (compared to children who were not overweight). Girls were more likely to be unfit but not overweight.

**Table 2:** **Demographic characteristics of groups stratified according to fitness and weight category**

Variable	Fit Recommended weight (N = 586)	Fit Overweight (N = 185)	Un-Fit Recommended weight (N = 155)	Un-Fit Overweight (N = 144)
Proportion Deprived/Affluent (45.5% (95% CI: 42.5%, 48.5%))	41.0% (37.0%, 45.0%)	49.7% (42.5%, 56.9%)	51.0% (43.1%, 58.9%)	52.8% (44.6%, 61.0%)
Proportion female (41.7% (95% CI: 38.7%, 44.7%))	38.4% (34.5%, 42.3%)	34.1% (27.3%, 40.9%)	60.0% (52.3%, 67.7%)*	45.1% (37.0%, 53.2%)
Mean Birth Weight (3.5 kg (95% CI: 2.8, 4.0))	3.4 (2.8, 4.0)	3.5 (2.8, 4.2)	3.4 (2.8, 4.0)	3.5 (2.9, 4.2)
Proportion Illness Affecting Exercise (10.5% (95% CI: 8.7%, 12.3%))	8.2% (6.0%, 10.4%)	13.7% (8.7%, 18.7%)	11.0% (6.1%, 15.9%)	14.5% (8.7%, 20.3%)
Proportion Receiving Medical Treatment (13.0% (95% CI: 11.0%, 15.0%))	10.8% (8.3%, 13.3%)	18.6% (13.0%, 24.2%)	10.4% (5.6%, 15.2%)	18.1% (11.8%, 24.4%)
Proportion Heart Disease in Family (40.8% (95% CI: 37.9%, 43.7%))	40.6% (36.6%, 44.6%)	41.9% (34.8%, 49.0%)	38.9% (31.2%, 46.6%)	43.2% (35.1%, 51.3%)
Proportion Diabetes in Family (45.0% (95% CI: 42.0%, 48.0%))	44.2% (40.2%, 48.2%)	45.3% (38.1%, 52.5%)	44.8% (37.0%, 52.6%)	48.1% (39.9%, 56.3%)
Proportion Stroke In Family (23.5% (95% CI: 21.0%, 26.0%))	22.9% (19.5%, 26.3%)	21.2% (15.3%, 27.1%)	23.0% (16.4%, 29.6%)	28.6% (21.2%, 36.0%)
Proportion High BP in Family (56.2% (95% CI: 53.2%, 59.2%))	54.8% (50.8%, 58.8%)	52.4% (45.2%, 59.6%)	59.5% (51.8%, 67.2%)	60.0% (52.0%, 68.0%)
Proportion High Chol in Family (41.9% (95% CI: 38.9%, 44.9%))	41.3% (37.3%, 45.3%)	43.6% (36.5%, 50.7%)	41.7% (33.9%, 49.5%)	43.0% (34.9%, 51.1%)
Proportion Smoker in Family (47.8% (95% CI: 44.8%, 50.8%))	46.9% (42.9%, 50.9%)	52.3% (45.1%, 59.5%)	50.8% (42.9%, 58.7%)	42.9% (34.8%, 51.0%)
Proportion Mother Obese (17.8% (95% CI: 15.5%, 20.1%))	12.6% (9.9%, 15.3%)	30.1% (23.5%, 36.7%)*	18.0% (12.0%, 24.0%)	23.1% (16.2%, 30.0%)*
Proportion Father Obese (26.1% (95% CI: 23.5%, 28.7%))	23.2% (19.8%, 26.6%)	34.4% (27.6%, 41.2%)*	23.3% (16.6%, 30.0%)	30.9% (23.4%, 38.4%)
Proportion having Vaccination (47.9% (95% CI: 44.9%, 50.9%))	50.3% (46.3%, 54.3%)	38.9% (31.9%, 45.9%)	45.8% (38.0%, 53.6%)	52.1% (43.9%, 60.3%)
Proportion FSM Eligible (25.9% (95% CI: 23.3%, 28.5%))	22.6% (19.2%, 26.0%)	31.9% (25.2%, 38.6%)	25.6% (18.7%, 32.5%)	31.0% (23.4%, 38.6%)
Proportion Achieve KS1 Test (83.9% (95% CI: 81.7%, 86.1%))	88.7% (86.1%, 91.3%)	80.6% (74.9%, 86.3%)	80.0% (73.7%, 86.3%)	76.1% (69.1%, 83.1%)
Proportion Achive KS2 Test (81.9% (95% CI: 79.6%, 84.2%))	84.5% (81.6%, 87.4%)	83.2% (77.8%, 88.6%)	75.0% (68.2%, 81.8%)	76.0% (69.0%, 83.0%)
Proportion Achive KS3 Test (78.4% (95% CI: 75.9%, 80.9%))	79.9% (76.7%, 83.1%)	75.9% (69.7%, 82.1%)	77.8% (71.3%, 84.3%)	74.1% (66.9%, 81.3%)
Proportion Large Gest (21.7% (95% CI: 19.2%, 24.1%))	20.6% (17.3%, 23.9%)	21.0% (15.1%, 26.9%)	24.5% (17.7%, 31.3%)	23.9% (16.9%, 30.9%)
Proportion Large 6 m – 12 m (19.3% (95% CI: 16.9%, 21.7%))	13.6% (10.8%, 16.4%)	32.2% (25.5%, 38.9%)*	21.6%(15.1%, 28.1%)	23.4% (16.5%, 30.3%)*
Proportion Large 12 m – 5 y (14.0% (95% CI: 11.9%, 16.1%))	7.4% (5.3%, 9.5%)	23.0% (16.9%, 29.1%)*	10.3% (5.5%, 15.1%)	33.8% (26.1%, 41.5%)*
Proportion High BP (11.1% (95% CI: 9.2%,13.0%))	8.7% (6.4%, 11.0%)	16.5% (11.2%, 21.8%)*	8.1% (3.8%, 12.4%)	16.4% (10.4%, 22.4%)
Proportion High Cholesterol (13.0% (95% CI: 11.0%, 15.0%))	11.3% (8.7%,13.9%)	12.6% (7.8%, 17.4%)	11.0% (6.1%, 15.9%)	21.8% (15.1%, 28.5%)
Mean Triglyceride level (0.8 (95% CI: 0.3, 1.2))	0.7 (0.4, 1.0)	0.8 (0.4, 1.2)	0.7 (0.4, 1.0)	1.0 (0.3, 1.8)
Proportion High Chol: HDL Ratio (4.5% (95% CI: 3.3%, 5.7%))	1.3% (0.4%, 2.2%)	7.6% (3.8%, 11.4%)*	3.1% (0.4%, 5.8%)	15.2% (9.3%, 21.1%)*
Proportion High Glucose (3.7% (95% CI: 2.6%, 4.8%))	2.6% (1.3%, 3.9%)	7.1% (3.4%, 10.8%)	3.6% (0.7%, 6.5%)	3.5% (0.5%, 6.5%)
Mean Fasting Insulin (10.2 (95% CI: 2.3, 18.1))	8.3 ( 2.7, 13. 9)	13.5 (3.5 23.6)	9.1 (1.3, 16.8	14.2 (5.0, 23.5)
Mean CRP (1.2 (95% CI: -0.95, 3.4))	1.1 ( -1.4, 3.5)	1.5 (-0.3, 3.6)	1.0 (-0. 6, 2.5)	1.5 (-0.1, 3.0)
Mean Adiponectin (3887 (95% CI: 1528, 6247))	4,030 (1,578, 6,483)	3,460 (1,451, 5,469)	4,296 (2,010, 6,582)	3,498 (1,134, 5,862)
Proportion with Asthma (10.0% (95% CI: 8.2%, 11.8%))	8.4% (6.2%, 10.6%)	10.8% (6.3%, 15.3%)	9.7% (5.0%, 14.4%)	16.0% (10.0%, 22.0%)
Proportion Infection (21.7% (95% CI: 19.2%, 24.2%))	21.0% (17.7%, 24.3%)	18.9% (13.3%, 24.5%)	21.9% (15.4%, 28.4%)	27.8% (20.5%, 35.1%)
Proportion Tonsillitis (21.1% (95% CI: 18.7%, 23.5%))	21.0% (17.7%, 24.3%)	23.8% (17.7%, 29.9%)	20.6% (14.2%, 27.0%)	18.8% (12.4%, 25.2%)
Median Visit GP (44 (IQR: 41))	44 (39)	44 (47.75)	44 (45.75)	44 (35.25)
Median Visit Out-patients (1 (IQR: 4))	1 (4)	1 (4)	1 (3)	1 (4)
Median Visit In-patients (1 (IQR: 1))	1 (1)	1 (1)	1 (2)	1 (2)
Median Visit AE Major (0 (IQR: 0))	0 (0)	0 (0)	0 (0)	0 (1)
Median Visit AE Minor (0 (IQR:)	0 (0)	0 (0)	0 (0)	0 (0)

*Significantly different from the Fit/recommended weight category (p < 0.05).

Data mining cluster analysis using the IM’s cluster operator suggests that there were 3 clusters for risk of future diabetes or heart disease (Table 3). The first cluster (829/1070, 77% of children) is a ‘low risk’ category where children are not obese, are fit, have normal blood test results (low triglyerides, CRP, fasting insulin, cholesterol), low infection and tonsillitis rates, and average number of GP visits. The second cluster (175/1070, 16% of children) is an ‘invisible at risk’ category where children are not obese but are unfit, have higher blood markers especially fasting insulin, higher triglycerides, CRP cholesterol, higher blood pressure, fasting glucose, and less likely to achieve academically especially at age 14 (Key stage 3). The third cluster (66/1070, 6% of children) is a ‘visibly at risk’ category where children are obese and unfit, and have high blood markers (high trigyceride, CRP), more infections, tonsillitis, vaccinations and more GP visits, and lower educational achievement.

**Table 3:** **Cluster analysis based on fitness and weight catergories**

Variables	Total Population	Low risk (77.48%)	Invisible at risk (16.36%)	Visibly at risk (6.17%)
	N = 1,070	Homogeneity = 0.675 N = 829	Homogeneity = 0.698 N = 175	Homogeneity = 0.541 N = 66
Obese	17.5% (180/1,028)	14.6% (120/824)	21.6% (33/153)	52.9% (27/51)
Unfit	36.4% (299/822)	32.3% (219/678)	50.9% (54/106)	68.4% (26/38)
Triglyceride	0.77	0.73	1.37	1.15
CRP	1.23	1.17	2.07	2.03
Fasting Insulin	10.20	9.68	18.69	16.31
Adiponectin	3,887.38	3,882.20	3,832.58	4,063.90
High BP	11.1% (111/1,003)	8.6% (70/811)	21.2% (31/146)	21.7% (10/46)
High Chol	16.4% (114/877)	10.9% (90/825)	58.3% (14/24)	35.7% (10/28)
High Chol:HDL Ratio	4.5% (32/708)	3.3% (22/670)	25.0% (3/12)	26.9% (7/26)
High Glucose	3.7% (29/791)	3.0% (22/744)	12.5% (3/24)	17.4% (4/23)
Asthma	10.0% (107/1,070)	9.2% (76/829)	1.7% (3/175)	42.4% (28/66)
Infection	21.7% (232/1,070)	21.1% (175/829)	4.0% (7/175)	75.8% (50/66)
Tonsillitis	21.1% (226/1,070)	19.2% (159/829)	16.0% (28/175)	59.1% (39/66)
KS1 Test	83.9%	88.7%	74.8%	55.6%
KS2 Test	81.9%	84.5%	74.8%	64.9%
KS3 Test	78.3%	80.2%	62.5%	76.9%
Visit Out	3.47	3.07	4.03	7.00
Visit In	1.18	0.90	1.61	3.50
Visit GP	49.80	47.84	29.51	79.75

### Qualitative findings

Five main themes regarding engagement in physical activity among deprived children were identified from analysis of the focus groups, namely: cost, accessibility, self confidence, parental support, and a general apathy towards physical activity.

#### Cost

Providing basics such as school uniform and other essentials is a struggle for some deprived families and this makes it difficult for children to ask for extra non-essentials such as attending activities. The teenagers participating in focus groups did not have disposable income and this meant value for money was very important.

“my mum struggles quite a bit to get me stuff” – Girl

#### Accessibility

Most activities such as the skateboard park, climbing wall and the leisure centre are not situated in deprived areas and so will require a car or transport to get to and from the activity. Thus, is can be complicated and expensive to get to the activity. In addition, access to activites was made more difficut when they needed prior booking, some were closed at weekends, and many were so popular they become overcrowded and it was hard to use the facilities.

“There’s nothing really to do in our area so you have to travel” – boy

#### Self confidence/competence

There was a lack of basic skills, such as swimming or riding a bike, and consequently a heightened sense of the danger involved with some sports. This lead to worries about safety of the sports. Combining this lack of competence of basic sporting skills with the prominent issue of self-image during teenage years gave a lack of self-confidence with sports all together. In addition, with activity generally not being the norm amongst the teenagers, there was also an embarrassment factor if you were deemed to be doing an activity out of the ordinary.

“I can’t ride a bike…I fell off it like a couple of months ago and I won’t ride a bike since” – girl

“It’s like if you’re starting and if you like mess up, they all just like laugh at you” - boy

“I was doing it before, running round the street and I was like, ‘oh my God I really need to stop” but I can’t because everyone’s looking” – girl.

#### Parental support

Parental influence tended to play a major role; for money for activities, transport to activities, simply acceptance and providing consent to participate in the activity. Some parents reportly had reservations about the safety of activities or even about the safety of getting to and from the activity itself, whereas others just don't see physical activity as an effective use of time or money.

#### Apathy

Some of the children simply were not intrested in activity. Interviews with the teachers suggested there was a cyclical generational barrier being passed on from parents to children, so parent's potentially negative perceptions of sport and physical activity are being instilled in their children. Thus, not participating in sport or activity was seen as the norm for the young people and their families.

“I think what tends to happen is you have generation after generation, people growing up and staying the same place so the problems these children are facing were faced by their parents and their grandparents so they won’t be encouraging them to go and do anything, other than football, because that is what they (the parents) did” – teacher.

## Discussion

This study shows that being unfit in early adolescence (11–13 years) is associated with being deprived, being a girl, having obesity in the family, and not achieving in education. Education has a strong effect and female gender has an an important effect in non-deprived areas.

A group of children can be identified who could be called a ‘invisibly at risk group’ who are not obese but are unfit. These children do have high risk markers for high blood pressure, high cholesterol, high fasting glucose, high CRP and fasting insulin, all factors which cannot be easily measured but could be predicted from fitness tests. In addition, these children are not achieving as well as average in school and this is progressive with only 62% achieving the recommended standard at age 14 (Key Stage 3). This suggests they may have health problems in the future and poor career prospects.

Qualitative findings suggest that not only is the cost of the actual activity a problem for deprived children, but they have to pay more than affluent children as activities are located in affluent areas so deprived children need to also get transport to the activities, then need to overcome barriers from parents and peers who have a negative view of being active and they start with a low level of basic skills.

Several studies [31–34] have shown a positive relationship between increased physical fitness levels and academic achievement. It has previously been shown that participation in activities improves academic achievement [34] and in fact physical fitness is a better predictor of academic achievement than obesity [35]. This may be because of direct effects such as, sports participation may improve numeracy [36] or indirect effect such as an activities offering an improved friendship and social network base. However, our findings showed a temporal relationship that not achieving academically, especially for deprived children and for boys, is a very strong predictor of being unfit in the future. Possibly academic achievement influences up take of public health messages, so children not doing well academically may then be high risk for ill health. However, this finding may also suggest that individuals who do not have support with their homework also do not have support to be active or healthy. Perhaps fitness can be used to highlight children where parenting skills and support (engaging with child including doing homework, talking, being active as a family) may improve the future for specific children [37] and academic achievement can be used to highlight those children who need extra interventions for health promoting behaviours. This study finds that children who are unfit are more likely to attend a school in a deprived catchment area. These schools would be ideal settings to implement interventions targeting adolescent health. For example, a number of strategies encompassing physical education, class room activities, extra-curricular sports and active transportation have been shown to be effective in increasing activity [38]. Leading on from the qualitative findings of this study, strategies need to include community wide campaigns introducing social support of physical activity, creation of improved access to places for activity and improved land use in existing deprived areas [38].

### Limitations

This study can only look at the children who took part in the fitness assessment. Although we did not find a difference between the sampled children and the class average fitness taken from the physical activity lessons, it is likely that non-participants may differ in characteristics such as ethnicity, obesity and family behaviours, which will mean that some of the associations identified in this study may be underestimated. We had an overall participant rate of 38% and given this study involved blood test in children aged 11–13 this was better than we expected. However, the lower participation was predominantly in the deprived and high ethnic minority schools suggesting the findings from this study may be an underestimate.

We found that children who under-achieve academically at ages 7 and 11 (Key Stages 1 and 2) were more likely to be un-fit at ages 11–13, but we know nothing about fitness levels in these children prior to them sitting their Key Stage tests. Thus we cannot comment on any cause-effect process in this relationship. Similarly we cannot comment on any temporal effect between fitness and health outcomes. Future studies should perform serial measure of fitness starting in early childhood to begin to establish the direction of causality between fitness and academic achievement.

Finally, we only present findings on interviews with children in a deprived school. We have previously published on focus group interviews with affluent and deprived children [39] but wanted to focus only on the barriers to activity among deprived children within this study in order to give context to the quantitative findings.

## Conclusion

This study found that girls, deprived children, those with obese parents and not achieving in education were more likely to be unfit. Achievement in education was the major factor among deprived children and female gender was the main factor among non-deprived children. Unfitness among non-overweight children could be used to highlight those individuals who are likely to have high cholesterol, high glucose and fasting insulin levels. Interventions need to focus on community change including improved access to activity in deprived areas, targeting parental support for being active and building competence and confidence in undertaking activity.

## Abbreviations

20MSFT: 20 metre multi stage fitness test
BMI: Body mass index
BP: Blood pressure
CRP: C-reactive protein
GP: General practitioner
IOTF: International obesity task force
IDF: International Diabetes Federation
IM: Intelligent miner
Chol: Cholesterol
FSM: Free school meal
KS1/KS2: Key stage 1/Key stage 2 (educational achievement tests)
Gest Age: Gestational age.
